# Medical Insights from Posts About Irritable Bowel Syndrome by Adolescent Patients and Their Parents: Topic Modeling and Social Network Analysis

**DOI:** 10.2196/26867

**Published:** 2021-06-09

**Authors:** Bu Zhong, Qian Liu

**Affiliations:** 1 Donald P. Bellisario College of Communications Pennsylvania State University University Park, PA United States; 2 School of Journalism and Communication National Media Experimental Teaching Demonstration Center Jinan University Guangzhou, Guangdong Province China

**Keywords:** irritable bowel syndrome, health care forum, adolescent, parents, topic modeling, social network analysis

## Abstract

**Background:**

Adolescents with irritable bowel syndrome (IBS) are increasingly seeking and sharing information about their symptoms in web-based health care forums. Their posts and those from their parents contain critical insights that can be used by patients, physicians, and caregivers to manage IBS symptoms.

**Objective:**

The aim of this study is to examine the posts from adolescent patients and their parents in a health forum, IBS Group, to better understand the key challenges, concerns, and issues of interest to young patients with IBS and their caregivers.

**Methods:**

Using topic modeling and social network analysis, in this study, we analyzed all the messages (over 750 topics and 3400 replies) posted on the IBS Group forum from 2010-2019 by adolescents with IBS aged 13-17 years and parents having children with IBS. We first detected 6 major topics in the posts by adolescent patients and parents on teenagers’ IBS symptoms and the interaction between the topics. Social network analysis was then performed to gain insights into the nature of web-based interaction patterns among patients and caregivers.

**Results:**

Using the Latent Dirichlet Allocation algorithm and a latent Dirichlet allocation visualization tool, this study revealed 6 leading topics of concern in adolescents with IBS: school life, treatment or diet, symptoms, boys’ ties to doctors, social or friend issues, and girls’ ties to doctors. The top 6 topics in the parents’ discussions were school life, girls’ issues, boys’ issues, diet choice, symptoms, and stress. The analyses show that the adolescent patients themselves are most concerned about the effect of IBS on their everyday activities and social lives. For parents having daughters with IBS, their top concerns were related to the girls’ school performance and how much help they received at school. For their sons, the parents were more concerned about the pain and suffering that their sons had to endure. Both parents and adolescents gained social support from the web-based platform. Topic modeling shows that IBS affects teenagers the most in the areas of pain and school life. Furthermore, the issues raised by parents suggest that girls are bothered more by school performance over pain, whereas boys show exactly the opposite: pain is of greater concern than school performance.

**Conclusions:**

This study represents the first attempt to leverage both machine learning approaches and social network analysis to identify top IBS concerns from the perspectives of adolescent patients and caregivers in the same health forum. Young patients with IBS must face the challenges of social influences and anxiety associated with this health disorder in addition to physical pain and other symptoms. Boys and girls are affected differently by pain and school performance and view the IBS impacts differently from the parents.

## Introduction

### Background

More than 58 million Americans are affected by irritable bowel syndrome (IBS) [1], a gastrointestinal disorder that causes abdominal pain, bloating, gas, and diarrhea or constipation. However, IBS in adolescents is a less known problem than it is among adults, although 6% of middle school students and 14% of high school students in the United States have symptoms indicative of IBS [2]. Patients with IBS, similar to others with chronic medical conditions, are increasingly seeking and sharing health information through web-based health care forums to manage their symptoms [3]. The forums, functioning similarly to online support groups, have served as a critical source of health information for patients and caregivers. On the internet, patients with gastrointestinal disorders could seek social, emotional, and peer support in addition to the real-time exchange of information related to medical concerns and symptom management [4].

Using data from the IBS Group forum, prior studies have investigated how the ways in which patients with IBS sought out or offered social support affected their behavior toward others on the forum [3,5]. For instance, expressing one’s emotions in a post during support seeking significantly increases the likelihood of receiving social support from others who are active in the web-based forum, but reciprocating support with a fixed set of peers discourages individuals from continued social support to others in the larger community [3]. User participation in the forum discussion was found to follow a power law, meaning that a small number of users on the forum became opinion leaders and generated most of the activity on the forum.

### IBS in Adolescents

IBS symptoms can be destructive to both adolescents and adults. Despite the serious impact of IBS on patients’ quality of life, this gastrointestinal disorder remains a clinical challenge that lacks diagnostic, radiologic, or laboratory markers [3]. To continue this stream of research, this research proposes to use probabilistic topic models for text summarization and topic detection by analyzing an unlabeled corpus of adolescent patients’ posts on their IBS symptoms and their parents’ posts on how their children coped with IBS. Specifically, we use the latent Dirichlet allocation (LDA) approach, a generative unsupervised probabilistic algorithm that isolates the top *K* topics in a data set, as described by the most relevant *N* keywords [6]. Thus, the posts and replies to them in the corpora are treated as random mixtures of latent topics, where each topic is characterized by a latent Dirichlet distribution over a fixed vocabulary. Here, the word *latent* means that topics must be inferred from keywords rather than directly observed.

Using the LDA topic modeling method [7], in this research, we first detected major topics in parents’ posts on the IBS Group forum discussing the IBS symptoms experienced by their teenaged sons and daughters. All the topics uncovered in the LDA analyses were analyzed and visualized to explore the relationships among the topics, whose results would assist in medical decision making for the teenagers, their parents, and health professionals. A social network analysis was also performed to gain insights into the nature of web-based interaction patterns.

The remainder of this paper is organized as follows. The next section provides the research background by reviewing the related literature. In the following section, we discuss the research questions (RQs) and explain the methodology. Subsequent sections present the main results and conduct a social network analysis. On the basis of the analyses, the findings and their implications, including the limitations, are examined in the *Discussion* section. Interventions have been proposed to mitigate IBS symptoms in adolescents. Finally, the conclusions are presented in the final section.

### IBS Diagnosis

IBS diagnosis is currently based on a symptom complex, and there is no definitive diagnostic test. In children and adolescents, the prevalence of IBS-associated abdominal pain ranges from 6% to 17% and 13% to 38%, respectively [2,8-10]. Previous studies of information flow on public patient-centered health care forums—namely, forums initiated and maintained by patients—have suggested that patients with IBS rely on health information on such forums to evaluate their medical decisions, health risks, and treatment options as well as seeking emotional support [11].

Children and adolescents are particularly vulnerable to IBS. Compared with adult patients, children and adolescents with IBS are distinct in the prevalence of IBS subtypes [12] and are typically prescribed with different pharmacologic and dietary management plans than adult patients [13]. Hence, physicians have difficulty in confirming their diagnoses [14] and may overlook some of the associations among multiple symptoms and facets of this health problem. The limited long-term treatment options deemed safe for younger patients, coupled with the intermittent and unpredictable recurrence of symptoms, result in a chronic state of uncertainty that has profound and formative impact on children’s and adolescents’ school performance and their quality of life, particularly in eroding younger patients’ self-esteem and magnifying their insecurity as *misfits* in schools and society. Although a large amount of work has been devoted to understanding IBS issues in adult patients, challenges in managing pediatric IBS symptoms are only beginning to garner the attention of researchers and health care practitioners.

Previous studies on adolescents’ IBS issues are helpful in understanding the pathophysiology of the disease and the etiology of abdominal functional pain, food-induced gastrointestinal symptoms, and other dietary consequences [2]. However, with rare exceptions [15], most studies did not consider possible complications that arise from the symptoms and everyday management of IBS during vulnerable developmental phases, such as childhood and adolescence. The novel aspects of our work are that it is the first study to leverage both machine learning approaches (eg, LDA [7,16]) and social network analysis to identify top IBS concerns from the perspectives of children, adolescents, and caregivers on web-based health forums. Such state-of-the-art approaches should be able to provide novel data-driven insights into factors that facilitate and impede the translation and uptake of health care knowledge and practices.

Therefore, this study proposed the following RQs to explore informational insights by analyzing the posts of adolescents with IBS and their parents who discussed the impact of IBS on young patients:

- RQ1. What are the top 6 topics in parents’ posts regarding the impact of IBS on their children?
- RQ2. What are the top 6 topics in teenagers’ posts on IBS symptoms?
- RQ3. Are there any sex differences between the concerns that parents post on the forum about their male and female children?
- RQ4. What is the nature of the interactions in the social network on the IBS health forum and what insights can we learn from it?

## Methods

### Sampling

In this study, we analyzed the topics in web posts from 2010 to 2019 from adolescents with IBS and the parents’ posts concerning the IBS symptoms experienced by their teenaged daughters and sons. The data for this study were retrieved in June 2019 from the subforum discussing *Teens and Children’s Issues* on the IBS Group forum [17]. All 734 topics in 3370 replies posted were collected and analyzed for adolescent patients. A preliminary analysis centers on identifying the topics when parents discussed the IBS issues experienced by their children aged between 13 and 17 years [18], involving 18 forum topics with 64 replies in this subforum. The LDA approach identified 6 major distinctive topics in their web-based discussion, as the analyses showed that if the number of topics was greater than 6, significant overlap could occur among them in the corpora.

### Social Network Analysis

**Figure 1:**
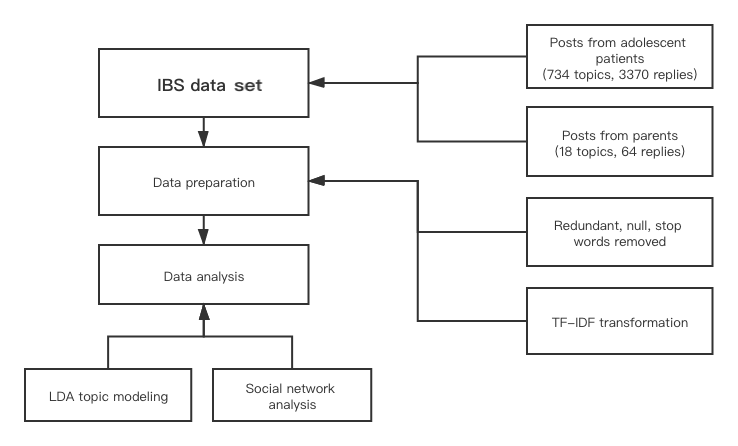
The process of data collection and analysis. IBS: irritable bowel syndrome; LDA: latent Dirichlet allocation; TF-IDF: term frequency-inverse document frequency.

For social network analysis, we parsed all posts to identify the links that arose when a pair of users participated in a discussion about a subject on the same thread. These links were further dissected to identify the most active users on the forum and the nature of the interactions among them. We also plotted the social network graphs for this data set and performed further analyses. To clean the data, we first removed the null and duplicate posts. Then, we also deleted common stop words such as *this*, *that*, *a*, and *the*. We first built a document-term matrix (eg, an N×M document-term matrix D is a matrix with N documents and M terms). We then applied term frequency-inverse document frequency to process the data so as to eliminate the weight for high-frequency words appearing in every document and add weight for words appearing only in a few documents. Scikit-learn and pyLDAvis libraries were then used to perform topic modeling analyses in Python. Figure 1 shows the data collection and analysis process.

## Results

### Overview

In this section, we first report the results from the *Parent’s Discussion* subforum and then those from the *Teens’ and Children’s Issues* forum.

### Parents’ Perspectives on Their Children’s IBS Issues

In the subforum of *Parent’s Discussion* under the *Teens and Children’s Issues* forum [18], we obtained 18 forum topics (or threads) and 64 replies after removing the duplicate, null, and invalid posts. A total of 82 posts were finally retrieved, including several comments from parents in the main forum: *Teens and Children’s Issues*. Hence, we wanted to separate the parents’ posts from those of the children. Parents’ posts usually refer to their children as daughters, sons, he, and she. By looking for such posts, it is possible to determine whether they were made by a parent or caregiver and also the sex of their children. On the other hand, posts by children or teenagers usually do not refer to their own sex and, with some exceptions, it is usually not possible to determine the sex of the person making the post. By searching for sex-specific keywords, we identified and manually transferred 455 posts from the main forum into the parents’ discussion subforum so that there were a total of 537 posts for analysis. The 6 topics that were discovered with 10 keywords each are listed in Table 1.

**Figure 2:**
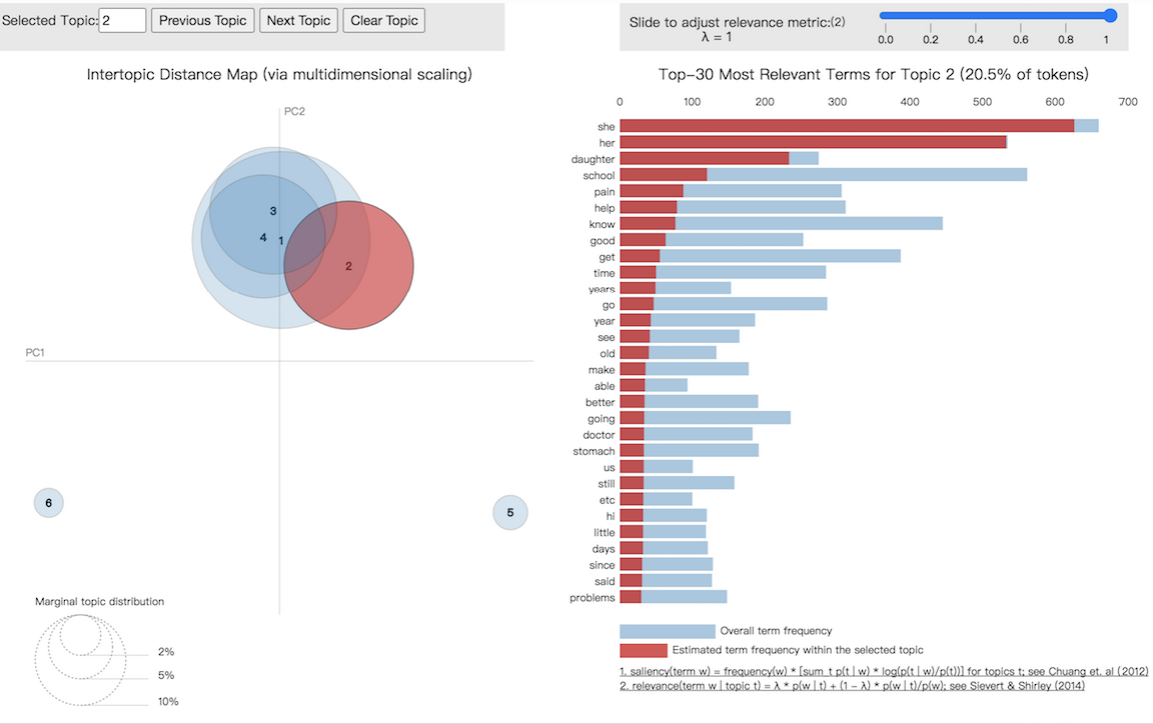
Latent Dirichlet allocation visualization of topic 2 (girls’ issues) in parents’ subforum.

**Figure 3:**
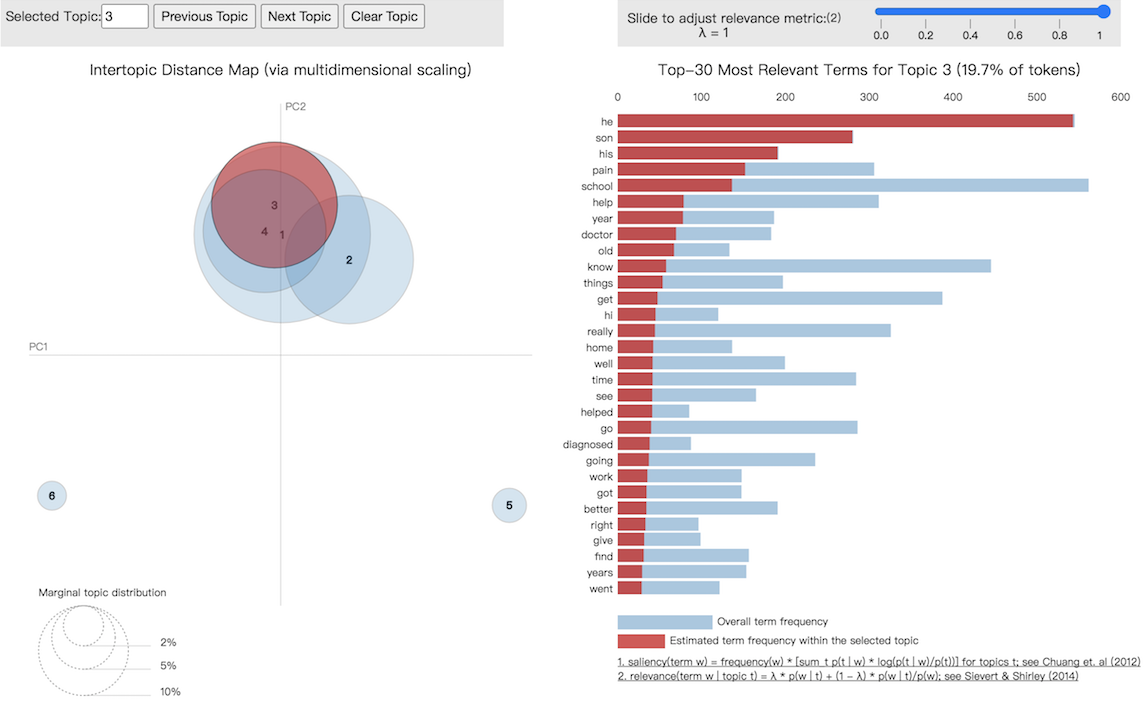
Latent Dirichlet allocation visualization of topic 3 (boys’ issues) in parents’ subforum.

We used the keywords to assign appropriate topic names to each detected topic. We also used the modified pyLDAvis library to generate an overview and visualization of the topic distribution regarding girls’ issues (Figure 2) and boys’ issues (Figure 3). In the figures, the sizes of the circles represent the relative importance of topics and the distances between the topics reflecting their similarity to one another [19].

**Table 1:** The top 6 topics in the parents’ discussion of their children’s irritable bowel syndrome issues.

Topic number	Topic^a^	Keywords
1	School life	*school*, *know*, *like*, *feel*, *people*, *think*, *time*, *stomach*, *want*, *tell*
2	Girls’ issues	*she*, *her*, *daughter*, *school*, *pain*, *help*, *know*, *good*, *get*, *time*
3	Boys’ issues	*he*, *son*, *his*, *pain*, *school*, *help*, *year*, *doctor*, *old*, *know*
4	Diet choice	*foods*, *diet*, *like*, *eat*, *food*, *try*, *lot*, *gas*, *good*, *help*
5	Symptom	*person*, *she*, *say*, *everything*, *problem*, *better*, *life*, *week*, *never*, *symptoms*
6	Stress	*worse*, *think*, *keep*, *diet*, *stress*, *pain*, *bad*, *say*, *started*

^a^The topic order is based on the total number of posts and replies each topic had from 1 (highest) to 6 (lowest).

As shown in Figures 2 and 3, for parents, their adolescent girls’ and boys’ issues related to IBS fell into two major topic groups. The girls’ issues, according to keywords representing the topic, are closely related to their school performance. Ignoring the frequently used keywords such as *she*, *her*, *daughter*, the other keywords indicate that parents seem to worry most about *school behavior* and the need of their children for *help*. Parent concerns about school attendance also appeared in their discussion on the subforum. An example post is as follows:

I know where we live, the schools get paid by the state based on how many days the kids are in school. So, this is why they are so concerned about the missed days. If I were you I would get a note from the doctor and bring it down to the school in person. Give a copy to the Principal, the Teacher and the School Nurse and explain the situation to them. This should help both your daughter and the school. Hopefully they will become a little more sympathetic to your daughter’s needs and she will feel better about going to school overall. Don’t let them bully you. Best of Luck and I hope your daughter starts feeling better soon.

Furthermore, parental worries about their daughter missing classes appeared in the discussion, as indicated by the following post:

See if you can get a 504. I have a similar problem, and when I first asked to start a 504, the school counselor said she is fine and doing well and doesn’t need one. She has not gotten into trouble for missing school, however, it causes both of us a lot of stress. I will be working on getting her a 504 next week so she will have it in place for high school. (A 504 is a plan for students who don’t qualify for an IEPER but still have “disabilities” that impair their normal every day activities.)

In contrast to the parents’ worries about their daughters’ school performance, for their sons, they appeared to be most concerned about their experience of pain and other symptoms brought on by IBS. This type of reaction can be seen in the discussion through posts such as the following:

My boy had a very strong reaction to the fructose solution. But none to the lactose. This was the only test in the long litany of tests around this miserable thing we call IBS that actually came out with an abnormal result. As a result he is on a low fructose and no fructan (fructans are in wheat and in the onion family especially). He has Post infectious IBS which was very severe during the first year of having it (he was treated by a chronic pain team in the end). Currently he has months when he is OK, normal really then the bowel problems always come back after a virus involving a sore throat.

Some other examples of parents discussing treatment of their child’s pain problems are as follows:

He is now on a very limited diet but his symptoms are so much better. He no longer has diarrhea or constipation on a regular basis, his stomach is no longer distended and best of all he hardly ever complains of stomach pains. We were also able to take him off all medications which treated symptoms but were not treating the cause of the pain. I suggest you see a good GI and have her tested for lactose intolerance and fructose malabsorption through Breath test and consider and endoscopy. Hope this helps and best of luck. I know it can be horrible to see your child in pain and feel you can’t do much about it.

My son has had for over 2 weeks pain in his stomach and usually in the middle and he goes to the bathroom but not a lot. It’s not hard and not really diarrhea but I think he has been backed up. He has had nausea and a headaches with this. He either is really hungry or not into eating at all.

Some other keywords also appeared in the discussion related to boys, such as *doctor* indicating parents’ excessive attention to doctors’ advice, diagnosis, and treatment, as it relates to boys. For example:

Now if your son is going to attend school great for him! I have a couple tips that might help him out with bathrooms etc. Go first to your doctor or specialist, have them fill out a form for IBS or a Doctor’s note that say the YES he does have IBS.

### Adolescent Patients’ Perspectives on Their IBS Issues

**Figure 4:**
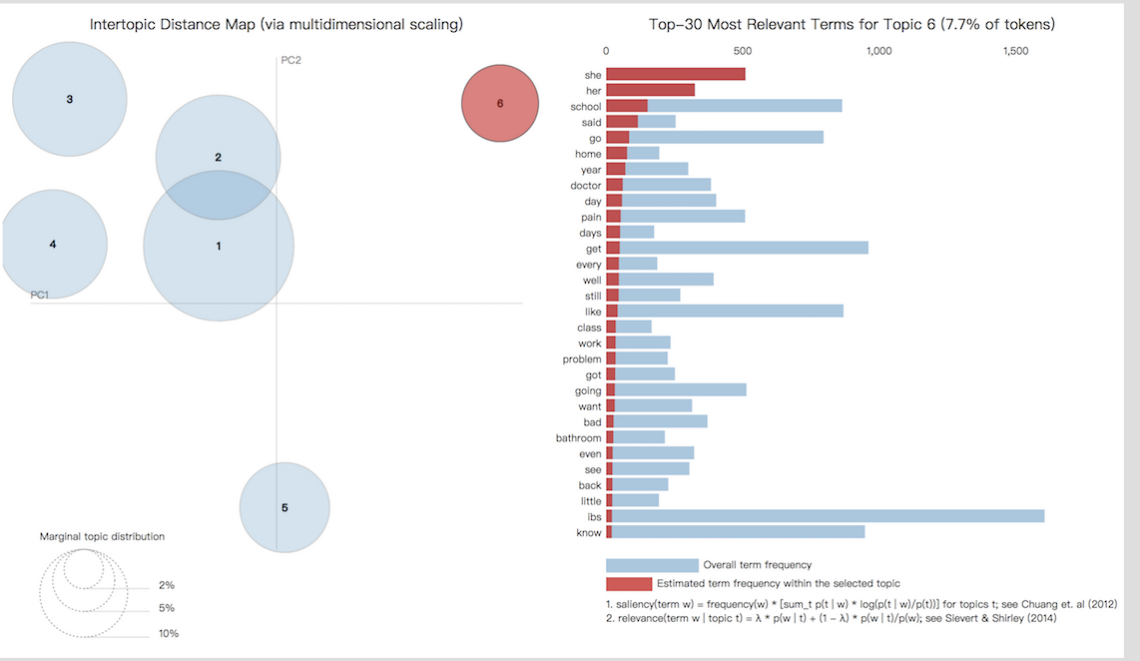
Latent Dirichlet allocation visualization of the top 6 topics in the posts from adolescent patients.

In the *Teens and Children’s Issues* forum [17], discussion occurred among the adolescents who were themselves the patients. We crawled 734 topics (or threads) with 3370 replies on this forum. After deleting the null and duplicate data, 2410 comments remained. Then, we removed the former 455 parents’ comments, which were transferred to the parents’ discussion; thus, the comments of the 1955 teenagers and children were left for further analysis. As done before, we cleaned the data and applied the LDA method. This generated 6 main topics, which are listed in Table 2. We also created visualizations for the topics as shown in Figure 4.

**Table 2:** The top 6 topics in the adolescent patients’ discussion of their irritable bowel syndrome issues.

Topic number	Topic^a^	Keywords
1	School life	*IBS*^b^, *school*, *know*, *life*, *try*, *think*, *hard*, *stress*, *want*, *need*
2	Treatment or diet	*eat*, *day*, *time*, *stomach*, *bad*, *IBS*, *sick*, *think*, *lot*, *little*
3	Symptoms	*IBS*, *pain*, *symptoms*, *diet*, *try*, *foods*, *help*, *food*, *find*, *thanks*
4	Boys’ ties to doctors	*he*, *help*, *doctor*, *feel*, *need*, *pain*, *know*, *IBS*, *try*
5	Social or friend issues	*friends*, *know*, *school*, *feel*, *talk*, *understand*, *time*, *hope*, *think*
6	Girls’ ties to doctors	*she*, *her*, *school*, *home*, *doctor*, *pain*, *class*, *work*, *problem*

^a^The topic order is based on the total number of posts and replies each topic had, ranging from 1 (highest) to 6 (lowest).

^b^IBS: irritable bowel syndrome.

As shown in Table 2, we concluded based on the LDA topic modeling analysis that there were 6 main topics in the posts. The first topic was calculated with keywords such as *IBS, school, know, life, try, think, hard,* and *stress.* We named this topic *school life*. The second topic was calculated with keywords such as *eat, day, time, stomach, bad, IBS, sick,* and *think,* and we named this topic *treatment or diet*. The third topic was calculated with keywords such as *IBS, pain, symptoms, diet,* and *try foods*, and we named this topic *symptoms*. It should be noted that schools, symptoms, and treatments were the most mentioned topics.

Among the symptoms, keyword frequencies showed that *stomach* and *pain* issues are the most devastating and irritating symptoms bothering patients in their daily lives. It is evident that for proper treatment, *food* and *diet* must be carefully planned. Eating more or less, quantity and diet choices are most frequently mentioned as ways to alleviate the problem in the diet options–related discussion. However, different patients have very personalized situations that they must pay attention to. Some discussed whether chocolate is suitable for patients with IBS, for example:

I feel your pain on not eating chocolate, but I’m so used to not getting to eat good food’ anymore that I just kind of pass right by it and don’t notice. Others concluded, on no junk food, for example: “no junk food, fried foods, dairy products, chocolate, soda...nothing!” Some patients seek help from doctors, some get advice from nutritionist, suggesting that “Often times it’s your diet, and a nutritionist can really help you in figuring out what foods are causing these problems.”

School performance was also an important topic for young patients and a cause of stress. They encountered difficulty in *fitting* into society, some mentioning experiences such as:

In high school I missed 1.5-2 days a week, and it was even more embarrassing because people noticed I frequently took days off and when I did show up the teachers, students used to brazenly point out my attendance (good to see you! followed by laughter). I avoided school even more. I wanted so desperately to graduate and not be forced to sit in a classroom.

Evidently, it was hard for them to be understood by their peers and teachers in school, with some complaining as follows:

Missing school is the worst as I know that nobody really understands what I’m going through.

One theme consistent in both the parents’ and teens’ forums is that although both worried and suffered stress or anxiety from IBS, their emphases differed. For parents, the stress was mostly related to IBS symptoms, such as pain, whereas for teenagers and children, it came from school and social activities. This means that parents should be encouraged to become more closely involved in the psychological and social impact of IBS on their children’s daily lives.

### Social Network Analysis

The purpose of the social network analysis was to understand the patterns of interaction among the users on this forum and extract insights from them. An interaction occurs between a pair of users when both are involved in the same thread by making posts related to the same topic, say, in response to a question raised on the forum. We found that there were 3010 unique interactions in all the posts on the *Teens and Children’s Issues* forum, including the parents’ subforums. After excluding the responses by the same author who created the thread, there were 2053 interactions. On examining the data more closely, we found that user *Kathleen M,* one of the top content contributors, was an IBS expert who also participated in the IBS forum for adults.

**Figure 5:**
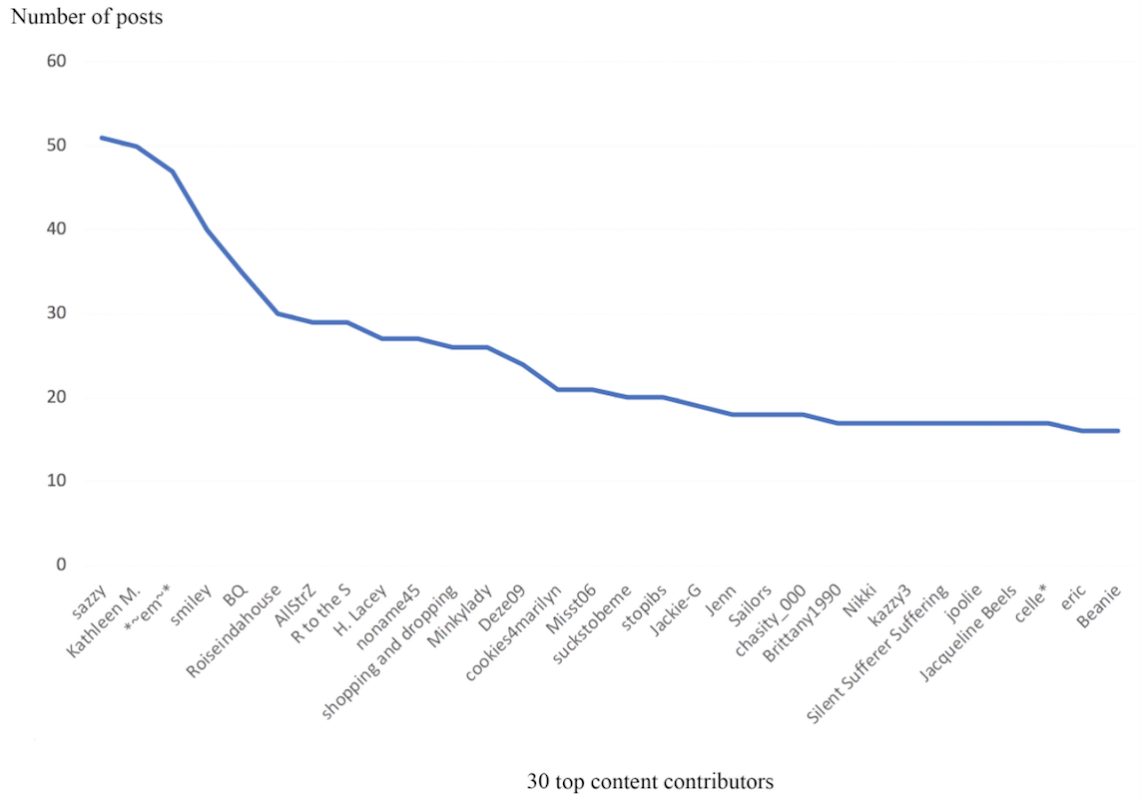
Plot of number of posts made by the 30 top content contributors.

Next, we plotted the top 30 most active users who contributed the most to the forum (Figure 5). The plot shows a typical power law pattern, where most of the contributions come from a small number of users and the participation of the remaining users is very small. This is also consistent with the pattern observed for the adult forum in our previous study [20,21].

**Figure 6:**
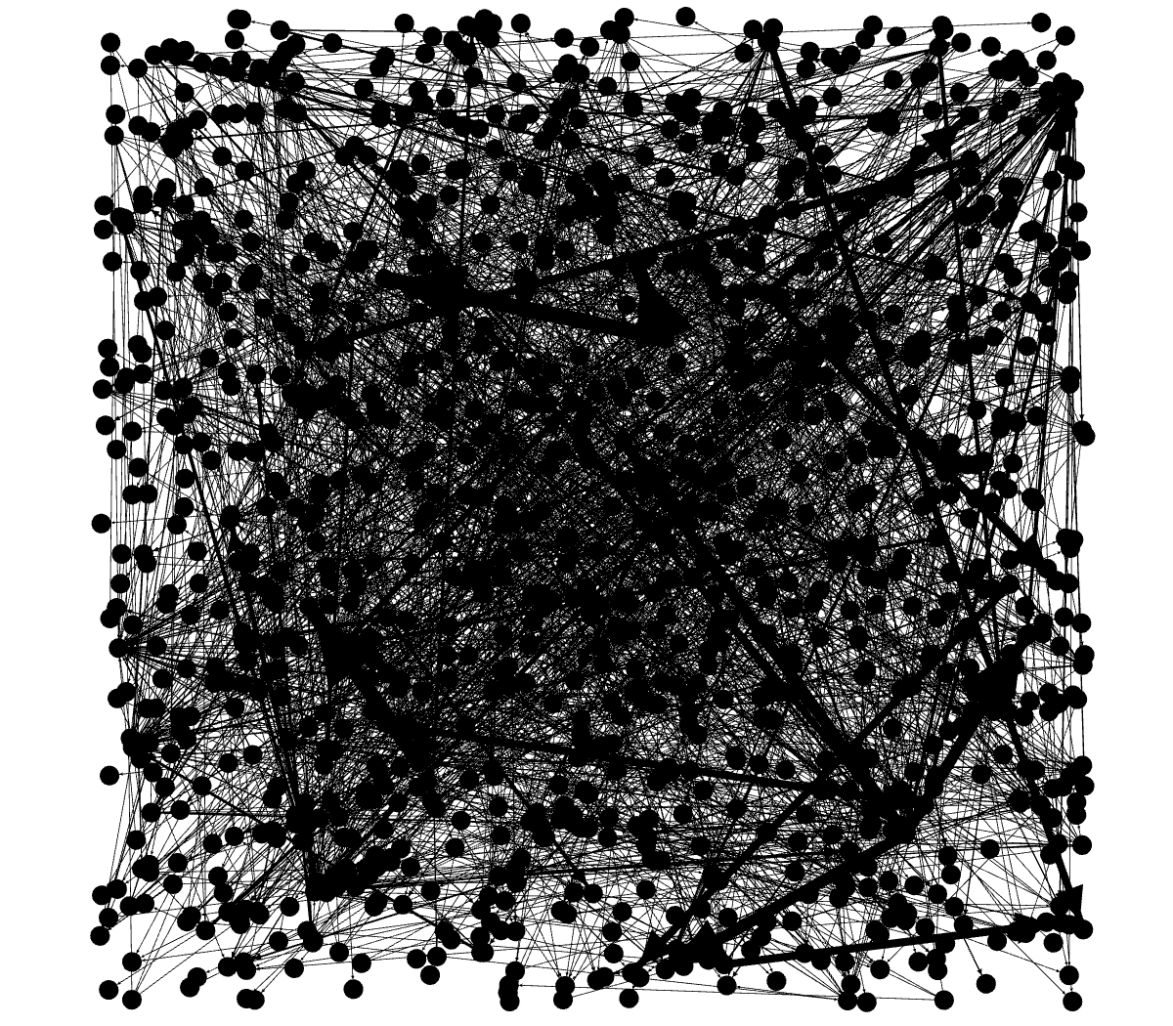
A full social network for all 2053 interactions on the forum.

Next, we used the Gephi tool [22] to create a social network graph of all interactions (Figure 6). This graph shows that there is a strongly connected core at the center from which there are connections radiating to the other nodes. The nodes on the outside have weak connections with each other. In this graph, there are 1165 nodes and 2020 edges. The edges are weighted by the number of exchanges that occur between the pair of users on the 2 sides. The weights vary from 1 to 5. The graph is undirected, so an edge with weight 1 represents the flow of a message in both directions. The density of the graph is 0.001, and its diameter is 16. The average degree of this graph is 1.734, and the weighted degree is 2.411. The clustering coefficient is 0.034.

**Figure 7:**
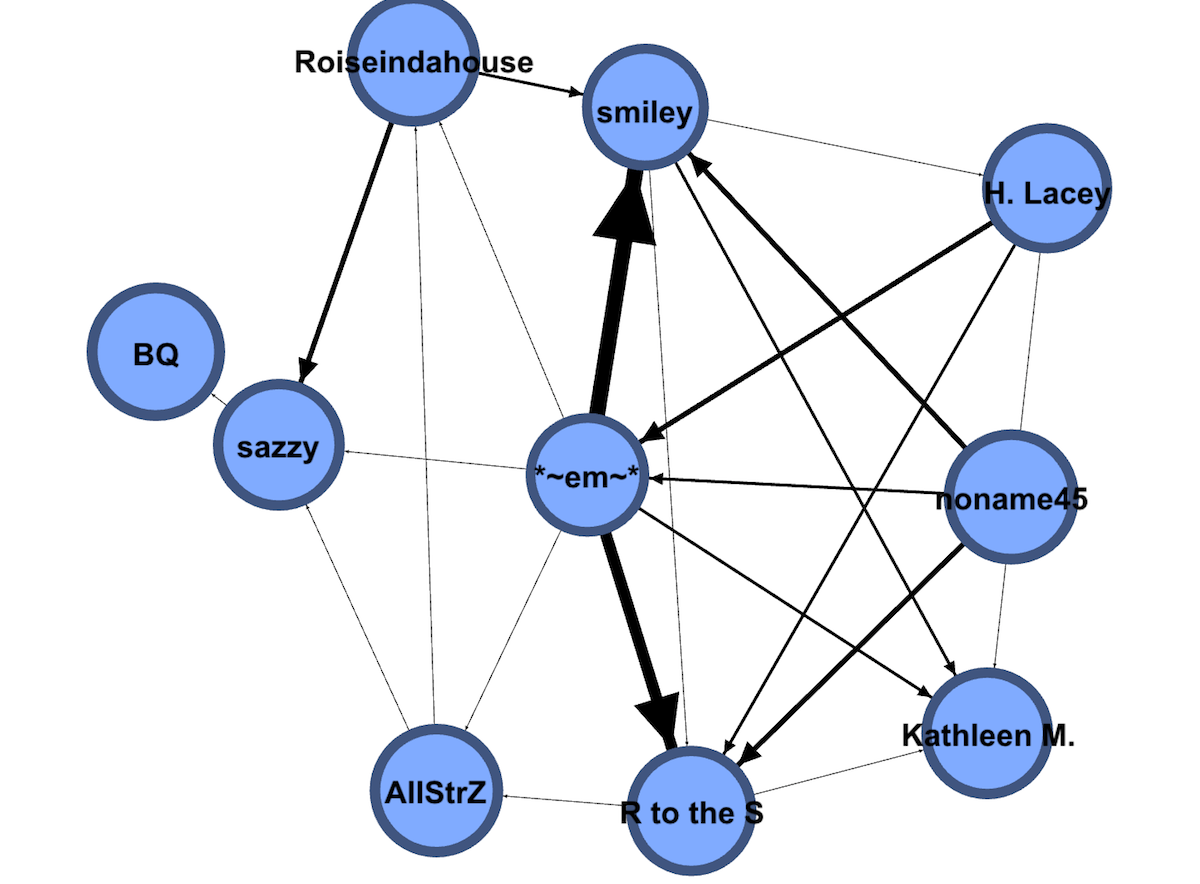
The social network of users with high-degree nodes (degree >25).

We also show a smaller filtered version of this graph in Figure 7. We deleted self-connected edges and showed a graph with nodes of degree >25 using the filter feature of Gephi to focus on the links among the most prolific contributors. The degree of a node in a social network is the number of connections it has to other nodes, and the degree distribution is the probability distribution of these degrees over the entire network, including both out degree, or the number of edges leaving a vertex, and in degree, which is the number of edges entering a vertex. The analyses show that this is a more tightly connected graph, as expected. The average degree of this graph and the average weighted degree are 2.6 and 4.867, respectively. The diameter is much smaller at 5, and the density is higher at 0.186. The clustering coefficient is 0.233.

As Figure 7 shows, the top contributors to the content in the forum had more interactions among themselves than with all other users in the same network. Among the top contributors, *Kathleen M* was found to contribute the most. It is important to note that this user or node did not have the highest closeness centrality, which reflects the inverse of one node’s average distance to all other nodes [23] and is one of the most important centrality indices in a network for the nodes [24]. Rather, *Kathleen M* was the top commentator on posts of others who did not post any inquiries in the forum. Other top content contributors, such as *~em~* and *sazzy*, posted inquires and comments on others’ messages and had the highest betweenness centrality in the forum, which is a key measure of distance in a network for a node that is calculated as the number of shortest paths going through a node. For *~em~*, the degree is 62, the closeness centrality is 0.275, and the betweenness centrality is 11,816.665, whereas for *sazzy*, the degree is 54, the closeness centrality is 0.168, and the betweenness centrality is 9749.487. These users were the 2 nodes with the highest degrees, closeness centrality scores, and betweenness centrality scores, even higher than those of the top content producer *Kathleen M* (degree=50, closeness centrality=0, and betweenness centrality=0). The results show that *Kathleen M* replies to many posts but did not post in this forum section, whose out degree is 0 and in degree is 50.

## Discussion

### Principal Findings

This study has discovered that IBS can be a sensitive topic for younger patients, particularly adolescents who are already undergoing myriad other critical developmental changes that deter them from sharing their feelings and emotions with adults, including their parents, caregivers, and teachers. Given the increasingly dominant role played by web-based platforms as a source of information exchange in this digital age, especially among adolescents [25], it is critical to harness information from web-based patient- and caregiver-centered forums to better understand key challenges, concerns, and issues of interest to younger patients with IBS and their caregivers; examine how health information, including misinformation and obsolete health care practices, is propagated and perpetuated via web media; and how the rates of information propagation and dissemination depend on the sentiments underlying health-related messages and links among forum members.

To date there is no definite cure for IBS, and hence, health care forums for IBS are very helpful to adult patients in exchanging medical information about their conditions and obtaining emotional support from others. Our semantic analysis of the posts reveals that adolescent patients with IBS and their parents also benefit from raising their issues and concerns regarding a forum dedicated to them. Physicians often advise them to follow a diet low in fermentable oligosaccharides, disaccharides, monosaccharides, and polyols and avoid *trigger foods* [13]. It is also important for teenagers to learn how to manage pain and stress, which are known to worsen the symptoms [26].

Previous research has found that adult patients tend to focus on gastrointestinal discomfort in their posts, and the top three causes were psychological factors, food, and allergens [27]. Using the LDA algorithm and an LDA visualization tool, we identified and visualized 6 main topics in our study from 2 health forums, one for discussion among the parents who cared for teen patients with IBS and another for discussion among the adolescent patients themselves. For parents, a top concern was the school performance of their daughters and how much *help* they got at school. For their sons, they were more concerned about the pain and suffering they had to endure. Teenagers themselves are most concerned about the effect of IBS on their everyday activities and social lives.

Although both sexes faced worries and anxieties from IBS, boys and girls responded differently to IBS symptoms, especially abdominal pain. It is evident that both teenagers with IBS and their parents valued the social support they received from web-based health care forums enormously. Our findings should also assist medical professionals and school administrators in enacting better health policies and lead to sound decisions and higher care quality for patients with IBS. A better understanding of the impact of IBS on young patients’ social and school lives can provide powerful support to both adolescents’ caregivers in self-caring IBS symptoms. Such self-management techniques based on the information shared on health forums are known as *a web intervention*, which have positive outcomes for patients with IBS [28]. Our research adds fresh evidence of the effectiveness of such a web intervention.

Going beyond previous studies on patients with IBS that seldom analyzed sex differences, we identified three reasons for the sex differences that were observed on the forum. First, girls found it difficult to describe their IBS issues to others to seek help and support. Some even became more reticent and felt humiliated easily. For instance, one adolescent was asked in front of her parents whether she was pregnant. Second, some young women have been brought up by parents to follow social norms and expectations, more so than boys. Third, the tolerance of pain varies between females and males with girls being more capable of enduring pain than boys. The findings reveal that in addition to coping with IBS symptoms, young patients have to face the challenges of social influences and anxiety associated with this health disorder in addition to physical pain and other symptoms. Boys and girls are affected differently by pain and school performance, and their views are different from those of parents.

Our social network analyses have identified multiple prolific users in the forum who contributed most content, such as posts and replies, in the forum. They functioned like opinion leaders in the network that could strongly influence other users’ health information processing concerning IBS. Their posts and replies could be the major drivers that kept the conversation of adolescent IBS symptoms going among the users, which also added value to the information flow. It is important to note that this study has found that one of the top content contributors such as *Kathleen M* did not post any messages but commented and replied to others’ posts. Without the social network analysis, it was impossible to know that the top content contributor achieved the status by writing replies and comments only, who was more influential than many other users who posted inquires and comments about IBS. Other influential users in this network, such as users *sazzy*, *~em~*, and *smiley*, posted inquires and commented on messages from their peers. This finding has important implications for users and caregivers when they exposed, accessed, and accepted the health information circulated in the forum. Without this study, average users would not know the veiled features of the information flow from the top contributors in the health forum.

Several limitations must be considered when interpreting these findings. First, the results may not be representative of the entire population of adolescents with IBS even when this study analyzed all the posts and replies from adolescent patients and parents on the IBS Group forum. Second, we cannot recognize the sex of the parents based on their posts and can only indirectly identify some of the adolescents’ sex by pronouns used, which may not be completely accurate. Third, because this is an anonymous forum, little information can be collected about the users, such as age, school year, health history including diagnoses, medicines, tests, allergies, immunizations, and treatment plans. All these factors make it difficult to fully understand their medical conditions, and thus, only limited insights may be revealed.

### Conclusions

IBS among children and teenagers is an underappreciated problem in the medical and school communities, although about 1 of every 7 adolescents suffers from it. This research uses a novel machine learning approach called the LDA algorithm to perform topic analysis on 2 forums—one for parents and another for teens to discuss their IBS problems. One of the important findings is that certain IBS concerns in teenagers are sex specific, which is not so apparent among adult patients. Parents have different issues that bother them for their sons or daughters. Some issues among girls and boys who suffer from this condition are also different. Both parents and children derive considerable medical and emotional benefits from IBS forums. Our study shows that this condition is very debilitating for adolescents, and in many cases, it has a devastating effect on their everyday activities and social lives with potentially lifelong consequences. This study represents the first attempt to leverage both machine learning approaches and social network analysis to identify top IBS concerns from the perspectives of adolescent patients and caregivers on the same web-based health forums. The insights revealed in this study, especially the social influences of IBS-related concerns, should help adolescent patients manage their symptoms and school lives. Moreover, caregivers, teachers, and school administrators should also benefit from the findings of this study.

## Abbreviations

IBS: irritable bowel syndrome
LDA: latent Dirichlet allocation
RQ: research question
